# Ethyl Chloride Narcosis and a New Apparatus for Its Proper Administration

**Published:** 1906-02

**Authors:** 


### ETHYL CHLORIDE NARCOSIS AND A NEW APPARATUS FOR ITS PROPER ADMINISTRATION.

In spite of all the advancement that has been made in the production of surgical narcosis the medical profession has constantly felt the need of safer, quicker and simpler methods of anaesthesia for general and emergency use. The apparatus about to be described would seem to meet these needs, and it is certain that it presents possibilities that cannot fail to appeal to every general practitioner. The administration of an anaesthetic is by no means the least important part of any operation, however grave and many times the skill of the surgeon comes to naught from the inefficient administration of ether or chloroform.

Recently ethyl chloride has been brought prominently forward as an ideal general anaesthetic, inasmuch as it has practically no contra-indications and possesses certain advantages that are indisputable. For some time objections were found in the methods of administering ethyl chloride, but these have been removed by the following new inhaling apparatus.

The inhaler consists of a face piece of soft flexible rubber, a connecting metal tubing, an inlet tube for the ethyl chloride, and a flexible rubber bag holding about one quart. The whole can be folded into small compass for the obsterical or surgical bag. With this apparatus and the employment of the simple directions, general anaesthesia can be produced with absolute safety, and the patient is not only rapidly and perfectly narcotized, but he recovers in a remarkable short time and presents no after-effects.

The name of the new inhaler is the ANTIDOLORIN INHALER, and the following can be said for it:

1. Absolute safety. 2. Ease of administration. 3. Rapidly produces surgical anaesthesia. 4. Can be used where chloroform or ether would be contradicted. 5. Patient can be kept in any position during anaesthesia, upright or prone. 6. No cyanosis occurs during administration, no swelling of the tongue, perfectly noiseless. 7. Patient recovers promptly without after-effects. 8. Inexpensive. 9. Can be used for long or short operations with equal success. 10. Especially useful as a preliminary to other anaesthetics, decreasing the time required for the production of anaesthesia and avoiding shock and discomfort to the patient.

The apparatus will be found of great utility by practitioners or surgeons who are called upon to do emregency surgical work. Its advantages will be apparent.

The chief advantage of this apparatus lies in the fact that the patient inhales a mixture of ethyl chloride and carbon dioxide, which appears to be more strongly anaesthetic than the ethyl chloride alone. Less of the ethyl chloride is needed, therefore, and moreover, none of it is wasted.

With ethyl chloride, ordinary precautions are required, as with nitrous oxide. The muscles are slightly contracted at the beginning but if the anaesthesia is pushed to a long period they relax. The respiration increases, pulsation slightly increases in number but not in force, higher centers appear stimulated (a distinct advantage) and the patient is not at any time syanosed. Children are excellent subjects; alcoholics poor ones.

Green thus describes the use of the apparatus in dentistry:

"Merely spray a small amount of the ethyl chloride into the admission tube, cover opening of same with your thumb, allow patient to take six or eight inhalations, then repeat this procedure until patient snores; when the stage for operation has begun, work leisurely, as patient will be sufficiently anaesthetized for a number of extractions. Three grammes of ethyl chloride usually suffice; five grammes is about the maximum required. A container with self-closing device is most preferable; if possible, have it graduated and with heavy spray, as this economizes time. Contraindications same as for nitrous oxide. Patient revives normally without after-effects; vomiting may occur but of no severity."

As a preliminary to ether or chloroform narcosis ethyl chloride has the same advantages over nitrous oxide as it has for use in minor operations.

